# Comparative Analysis of Color Space and Channel, Detector, and Descriptor for Feature-Based Image Registration

**DOI:** 10.3390/jimaging10050105

**Published:** 2024-04-28

**Authors:** Wenan Yuan, Sai Raghavendra Prasad Poosa, Rutger Francisco Dirks

**Affiliations:** Independent Researcher, Oak Brook, IL 60523, USA; raghava0807@gmail.com (S.R.P.P.); rutger.dirks@gmail.com (R.F.D.)

**Keywords:** comparison, image stitching, OpenCV, Python

## Abstract

The current study aimed to quantify the value of color spaces and channels as a potential superior replacement for standard grayscale images, as well as the relative performance of open-source detectors and descriptors for general feature-based image registration purposes, based on a large benchmark dataset. The public dataset UDIS-D, with 1106 diverse image pairs, was selected. In total, 21 color spaces or channels including RGB, XYZ, Y′CrCb, HLS, L*a*b* and their corresponding channels in addition to grayscale, nine feature detectors including AKAZE, BRISK, CSE, FAST, HL, KAZE, ORB, SIFT, and TBMR, and 11 feature descriptors including AKAZE, BB, BRIEF, BRISK, DAISY, FREAK, KAZE, LATCH, ORB, SIFT, and VGG were evaluated according to reprojection error (RE), root mean square error (RMSE), structural similarity index measure (SSIM), registration failure rate, and feature number, based on 1,950,984 image registrations. No meaningful benefits from color space or channel were observed, although XYZ, RGB color space and L* color channel were able to outperform grayscale by a very minor margin. Per the dataset, the best-performing color space or channel, detector, and descriptor were XYZ/RGB, SIFT/FAST, and AKAZE. The most robust color space or channel, detector, and descriptor were L*a*b*, TBMR, and VGG. The color channel, detector, and descriptor with the most initial detector features and final homography features were Z/L*, FAST, and KAZE. In terms of the best overall unfailing combinations, XYZ/RGB+SIFT/FAST+VGG/SIFT seemed to provide the highest image registration quality, while Z+FAST+VGG provided the most image features.

## 1. Introduction

Image registration is the computer vision task of aligning images of a common scene that differ due to their geometry or photometry conditions. Commonly, image registration is regarded as a component of a very close if not interchangeable concept, image stitching, which also involves image blending to create seamless stitched panoramas [1]. The core objective of image registration is to establish spatial correspondences between different images, allowing for the fusion of data from various sources or time points. Commonly, image registration algorithms are categorized into area-based and feature-based methods, although alternative classifications based on registered image types or image transformation types exist [2], given the complexity and diversity of different image registration approaches. Area-based methods rely on comparing and correlating pixel intensity patterns or statistical properties between corresponding image regions for optimization [3], while feature-based methods rely on detecting and matching landmarks or keypoints to estimate geometrical image transformations [4]. As researchers across all disciplines collectively embark on a new era of artificial intelligence, deep learning techniques have also been successfully applied to various image registration tasks such as feature extraction, descriptor matching, homography estimation, etc. [2,5,6].

Image registration has applications in diverse fields, such as art restoration [7], astronomy [8], geology [9], archaeology [10], oceanography [11], agriculture [12], remote sensing [13], materials science [11], medicine [14], robotics [15], augmented reality [9], military [16], etc. Despite the advancements in modern machine learning-based image registration algorithms, feature-based image registration, the concept of which dates back to at least the 1980s [17], still remains relevant even in recent literature, owing to its simplicity, efficiency, and robustness. As a few examples, Ramli et al. [18] proposed CURVE feature of retinal vessels to align fundus images; Nan et al. [19] utilized SURF and HC features for brain tissue image registration and analysis; Hou et al. [20] employed HC feature for panchromatic and multispectral satellite image alignment; Kerkech et al. [21] registered visible and infrared unmanned aerial vehicle (UAV) images through AKAZE feature for vine disease detection; Xue et al. [22] combined visible and infrared missile-borne images based on enhanced SIFT feature to improve target identification and striking; Wang et al. [23] proposed GOFRO feature to achieve high-precision synthetic aperture radar (SAR) image registration; and Bush et al. [24] used SIFT feature for bridge defect growth tracking.

Generally, the pipeline of feature-based image registration comprises several standard steps, while each step allows for variations in implementation. Given a pair of target and source images to be registered, the location of pixels of interest or keypoints are first detected based on feature detection algorithms or feature detectors. Example detectors include AKAZE [25], BRISK [26], CSE [27], FAST [28], HL [29], KAZE [30], MSD [31], ORB [32], SIFT [33], SURF [34], TBMR [35], etc. Next, the local neighborhoods of the detected keypoints are characterized based on feature description algorithms or feature descriptors. Example descriptors include AKAZE [25], BB [36], BEBLID [37], BRIEF [38], BRISK [26], DAISY [39], FREAK [36], HOG [40], KAZE [30], LATCH [41], LUCID [42], ORB [32], PCT [43], SIFT [33], SQFD [44], SURF [34], TEBLID [45], VGG [46], etc. Note certain feature detectors and descriptors can have the same names when they are proposed in the same studies. Based on the similarities between feature descriptions, which can be quantified through metrics such as Euclidean distance [42] or Hamming distance [47], the corresponding keypoints in the two images can be matched through feature description matching algorithms or descriptor matchers. Example matchers include brute-force (BF) [48], fast library for approximate nearest neighbors (FLANN) [48], k-nearest neighbors (KNN) [49], etc. Optionally, erroneous or unreliable feature matches can be filtered out based on cross-checking, which considers a match to be valid only when the two matched features from the two images both best-match with each other, or Lowe’s ratio test [33], which checks whether the feature distance of the best match is substantially smaller than that of the second-best match by a specified ratio threshold. The filtered feature matches are finally utilized to estimate homography, the transformation relationship between the source image plane and the target image plane to allow for image registration. Depending on the desired degree of freedom, or the level of source image warping, common 2D transformation types include rigid, similarity, affine, projective, etc. [50]. Numerous methods also exist for homography matrix calculation, such as least squares [51], least median [52], random sample consensus (RANSAC) [53], progressive sampling consensus (PROSAC) [54], etc.

Given the diversity of available feature detectors and descriptors for image registration, the question of selecting the most appropriate features inevitably arises. Although a handful of studies in current literature have investigated this topic, consensus cannot always be drawn from the study findings, unfortunately, due to the significant disparities in the experiment designs. Köhler et al. [55] manually annotated 28 evenly-spaced landmarks in a laparoscopic video with 750 frames as ground truth, comparing ORB, AKAZE, and BRISK detectors in combination with BEBLID descriptor based on reprojection error (RE) and structural similarity index measure (SSIM). Among the three detectors, AKAZE achieved the best mean and mean normalized REs, while BRISK achieved the best mean SSIM. Based on various public datasets, Tareen and Saleem [56] selected six image pairs of diverse scenes for feature number and speed evaluation, synthesized five pairs of ground truth images through resizing and rotating images by known levels for feature accuracy evaluation, and compared five features, including SIFT, SURF, KAZE, AKAZE, ORB, and BRISK, treating each as both detector and descriptor. They discovered that ORB detected the greatest number of features, while KAZE detected the lowest number; ORB had the lowest computation cost for feature detection and description, while KAZE had the highest; and SIFT was the most accurate feature for scale, rotation, and affine image variations overall. Sharma et al. [4] analyzed 85 detector and descriptor combinations based on three pairs of images, involving GFTT, SIFT, MSER, FAST, SURF, CSE, BRIEF, DAISY, AGAST, BRISK, ORB, FREAK, KAZE, AKAZE, and MSD. Their evaluation metrics included peak signal-to-noise ratio (PSNR), SSIM, feature similarity indexing method (FSIM), and visual saliency-induced index (VSI). AKAZE detector and AKAZE descriptor was identified as the best combination that outperformed all the other combinations. Wu et al. [57] compared SIFT with its variants PCA-SIFT, GSIFT, CSIFT, SURF, and ASIFT under scale and rotation, blur, illumination, and affine changes based on four pairs of images. They qualitatively concluded that SIFT and CSIFT performed the best under scale and rotation change; GSIFT performed the best under blur and illumination changes; and ASIFT performed the best under affine change. Ihmeida and Wei [49] created two datasets out of the same three remote sensing image pairs, and analyzed SIFT, SURF, ORB, BRISK, KAZE, and AKAZE as feature detector and descriptor simultaneously based on inlier match number, computation cost, and feature inlier ratio. They discovered that SIFT provided the highest accuracy, while ORB was the fastest algorithm.

In addition to the inconsistent conclusions on the best-performing feature detectors and descriptors, several knowledge gaps still exist in current literature regarding the general feature-based image registration procedure. First, before feature detection, color images are typically converted into grayscale for computation efficiency [58]. However, the value of color information contained in various color spaces pertaining to image registration has never been examined and quantified. Second, image registration quality is being evaluated using diverse methods in current literature, e.g., subject visual inspection and rating on parallax error, perspective distortion, viewing ease and comfort, etc. [59,60,61,62,63], as well as objective indices such as FSIM [4], mutual information (MI) [64], normalized cross correlation (NCC) [64], PSNR [4], RE [55], root mean square error (RMSE) [65], SSIM [4,55], stereoscopic stitched image quality assessment (S-SIQA) [66], universal image quality index (UIQI) [67], VSI [4], etc. Yet, the relationships or agreements between different evaluation metrics have not been investigated. Third, existing studies usually concentrate on quantifying image registration accuracy and speed when comparing various image features; however, feature robustness or reliability in successfully registering multiple image pairs without failure is a rarely discussed aspect. Finally, an effort comparing multiple image feature detectors and descriptors based on a large dataset is simply missing in current computer vision research, as many prior comparative studies tended to rely on only a few image pairs, potentially leading to biased conclusions.

To address the aforementioned lacunae in the existing knowledge base, the current study leveraged dedicated open-source dataset and library for image registration, with consideration given to practicality and replicability for future researchers. In terms of image registration accuracy, robustness, and feature numbers, the performance of selected color spaces and the corresponding color channels, feature detectors, and feature descriptors was quantified and presented in this article. Recommendations on the best overall combinations of color space or color channel, feature detector, and feature descriptor were also provided at the end of the study.

## 2. Materials and Methods

### 2.1. Dataset

**Figure 1:**
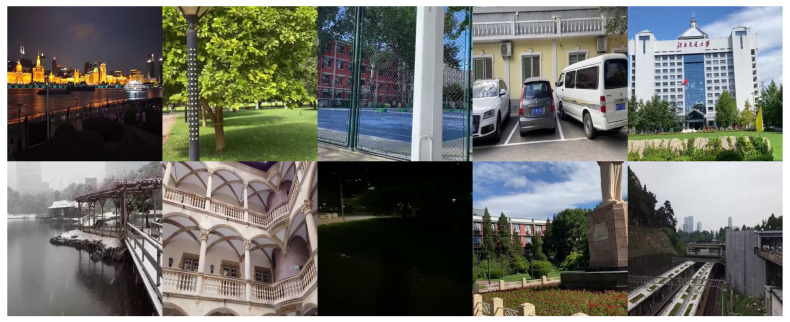
Sample images from the testing subset of the dataset UDIS-D [68].

The public dataset UDIS-D [68] was selected for the study due to its accessibility, substantial size, and image diversity. UDIS-D, proposed by Nie et al., is the first large real-world benchmark dataset for image registration, and it includes diverse scene conditions such as indoor, outdoor, night, dark, snow, zooming, etc., with different levels of image overlap and parallax. In particular, UDIS-D includes two subsets: a training subset containing 10,440 image pairs and a testing subset containing 1106 image pairs, which all have a 512 × 512 resolution. Only the testing subset was used in the current study, as it has a sufficiently large dataset size while preserving the same data diversity as the training subset (Figure 1).

### 2.2. Selected Color Spaces and Channels

**Figure 2:**
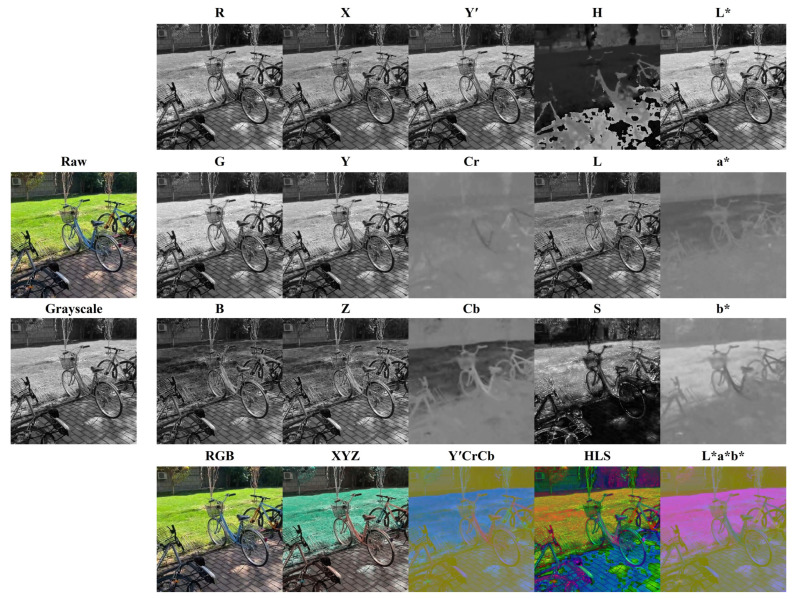
The investigated 21 versions of color space or channel of a sample image from the dataset.

Color space refers to a specific organization of colors, which allows for the representation of colors in a numerically and visually meaningful way. In feature-based image registration, image color information, most commonly represented by red–green–blue (RGB) color space, is usually discarded by converting RGB images into grayscale images for more efficient image feature detection and description. For the current study, five widely adopted color spaces in computer vision research were chosen to examine the value of color information under the context of image registration: RGB, XYZ, Y′CrCb, HLS, and L*a*b* [69]. For each color space, in addition to utilizing all matched features from all three color channels, the usefulness of individual color channels as potential superior replacements for standard grayscale conversion were also investigated, as color channels are grayscale images themselves. In total, each pair of the raw images from the dataset corresponded to 1 grayscale + 5 three-channel color spaces + 5 × 3 single color channels = 21 versions of color space or channel for feature detection, description, matching, and filtering during registration (Figure 2). All image color space conversion operations were completed using the open-source library OpenCV version 4.9.0 without any additional image processing steps before or after the conversions, the mathematical expressions of which can be found in [70].

### 2.3. Selected Detectors and Descriptors

The selection of image feature detectors and descriptors for the study was based on the following considerations, aimed at facilitating the replication of the study results and ensuring practical benefits from the study conclusions: the chosen detectors and descriptors should be implemented in open-source libraries; the chosen detector and descriptor functions should be stable for consecutive executions without raising fatal computer errors; the chosen detectors and descriptors should be freely available for use without patent protections; the chosen detector and descriptor functions should require no arguments to initialize the features; the outputs of the chosen detector functions should be compatible with the inputs of the chosen descriptor functions. Accordingly, nine feature detectors and 11 feature descriptors were selected (Table 1 and Table 2). Out of all the possible detector and descriptor function combinations, there were 15 never-compatible ones (Table A1). In total, 9 detectors × 11 descriptors − 15 incompatible combinations = 84 detector and descriptor combinations were investigated in the study. All image feature detection and description operations were completed using OpenCV version 4.9.0.

### 2.4. Image Registration Procedure

**Figure 3:**
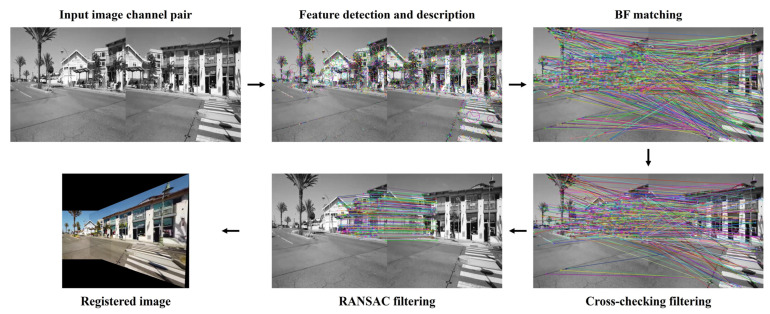
Schematic diagram of the employed image registration pipeline.

Before registration, each pair of raw images from the dataset was first converted to grayscale or the specified color space. Depending on whether the current registration involved single grayscale channel, single color channel, or all three color channels, the feature detection, description, matching, and filtering processes described below were repeated either once or three times. On the target image channel, image features were first detected by the specified feature detector and then described by the specified feature descriptor. The described image features were matched using BF descriptor matcher and filtered by cross-checking, as explained in the introduction. The binary descriptors, including AKAZE, BRISK, and ORB, were matched based on Hamming distance, while the non-binary descriptors, including BB, BRIEF, DAISY, FREAK, KAZE, LATCH, SIFT, and VGG, were matched based on Euclidean distance. If the current registration involved all three color channels, the three sets of filtered feature matches were combined as one set. Finally, projective homography was estimated based on the filtered feature matches through RANSAC, which could robustly filter out outlier feature matches that survived through cross-checking but did not agree with the majority (Figure 3). In total, 1106 raw image pairs × 21 color spaces or channels × 84 detector and descriptor combinations = 1,950,984 image registrations were performed in the study. All failed registrations or failed registration code executions were recorded, which could be due to intermittent detector and descriptor incompatibility, fewer than four inlier matched features after RANSAC for homography estimation, excessive registered source image distortion surpassing computer memory capacity from extreme homography transformation, etc. All image registration operations were completed using OpenCV version 4.9.0 in a Python 3.11.5 environment with default function argument values, unless specified otherwise above.

### 2.5. Registration Quality Evaluation

**Figure 4:**
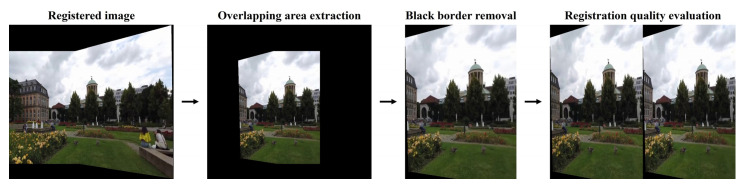
Image preprocessing steps before registration quality evaluation.

As ground truth registrations do not exist for the UDIS-D dataset, the current study evaluated the image registration quality based on the similarities between the overlapping areas of the target and source images, since perfect registrations should result in identical overlapping areas. While the image registrations were executed on multiple computers with different hardware specifications, registration speed was not considered as an essential aspect to evaluate for the study. Before any evaluation metrics could be properly calculated, preprocessing steps of the registered target and source images were necessary to ensure unbiased objective registration quality assessment. By default, OpenCV blends source image edge pixels with black background pixels during image warping to avoid artifacts and jagged edges, which, however, compromises original source image pixel values. When extracting target and source image overlapping regions, such edge pixels were specifically not counted as overlapping pixels. Additionally, OpenCV by default fills empty spaces with black background pixels in the registered images, which could affect certain metric calculations, although by a minimal amount. During target and source image overlapping region extraction, the black borders around the overlapping regions were removed as much as possible without sacrificing any valid overlapping pixels (Figure 4).

Three commonly used metrics were selected to objectively quantify the image registration quality in the study:

- $RE=\frac{1}{F}\sum_{i=1}^{F}\sqrt{{\left(x_{i}-{x_{i}}^{\prime }\right)}^{2}+{\left(y_{i}-{y_{i}}^{\prime }\right)}^{2}}$
  Where F is the number of inlier matched features after RANSAC homography estimation, (x_i_, y_i_) are the coordinates of the ith feature in registered target image, and (x_i_′, y_i_′) are the coordinates of the ith feature in registered source image. RE ranges from 0 to positive infinity.
- $RMSE=\sqrt{\frac{1}{3P}\sum_{x=1}^{W}\sum_{y=1}^{H}{\left(R_{x,y}-{R_{x,y}}^{\prime }\right)}^{2}+{\left(G_{x,y}-{G_{x,y}}^{\prime }\right)}^{2}+{\left(B_{x,y}-{B_{x,y}}^{\prime }\right)}^{2}}$
  Where P is the number of pixels in the overlapping area between registered target and source images excluding black background pixels, W is the overlapping area width, H is the overlapping area height, (x, y) are the overlapping area pixel coordinates, (R_x,y_, G_x,y_, B_x,y_) are the R, G, B values at pixel location (x, y) in registered target image, and (R_x,y_′, G_x,y_′, B_x,y_′) are the R, G, B values at pixel location (x, y) in registered source image. RMSE ranges from 0 to 255 for typical 24-bit images.
- $SSIM=\frac{1}{N}\sum_{i=1}^{N}\frac{\left(2\mu_{i}{\mu_{i}}^{\prime }+6.5025)(2\sigma_{c}+58.5225\right)}{\left({\mu_{i}}^{2}+{{\mu_{i}}^{\prime }}^{2}+6.5025)({\sigma_{i}}^{2}+{{\sigma_{i}}^{\prime }}^{2}+58.5225\right)}$
  Where N is the number of image patches where local SSIM is calculated within a 7 × 7 sliding window, μ_i_ is the mean of the ith patch in registered grayscale target image, μ_i_′ is the mean of the ith patch in registered grayscale source image, σ_c_ is the covariance of registered grayscale target and source images, σ_i_ is the variance of the ith patch in registered grayscale target image, and σ_i_′ is the variance of the ith patch in registered grayscale source image. SSIM ranges from −1 to 1. All SSIM values were calculated using scikit-image [71] version 0.20.0 with default function argument values.

## 3. Results and Discussion

### 3.1. Registration Quality Comparison

As shown in the following sections, RE tended to provide extremely large values when a low-quality registration was performed, unlike RMSE and SSIM, whose values were distributed within finite ranges. Perfect RE values such as 0 were achieved in the study; however, they were usually the result of low numbers of inlier feature matches after RANSAC filtering and hence could be misleading. For example, a homography estimated based on only four feature matches will achieve a perfect feature reprojection, which, however, is not necessarily equivalent to a high-quality registration, as the homography can represent an overfitted image transformation relationship. Additionally, the absence of large RE values did not necessarily indicate high-quality image registrations either, as the registrations simply could have failed. RMSE represents the average pixel value difference between registered target and source images. No perfect RMSE values such as 0 were achieved, which was anticipated, as the registration process generally would warp source images and distort their pixel values to some degree. All SSIM values in the study were larger than 0, indicating that the overlapping areas between the registered target and source images were always somewhat similar luminance, contrast, or texture-wise. Similar to RMSE, no perfect SSIM values such as 1 were achieved either. The large value ranges of the metrics reflected the diversity of registration difficulty within UDIS-D as an appropriate benchmarking dataset, including both easy registration, which would lead to low RE and RMSE values and high SSIM values, and difficult registration, which would lead to high RE and RMSE values and low SSIM values.

#### 3.1.1. Color Space

**Figure 5:**
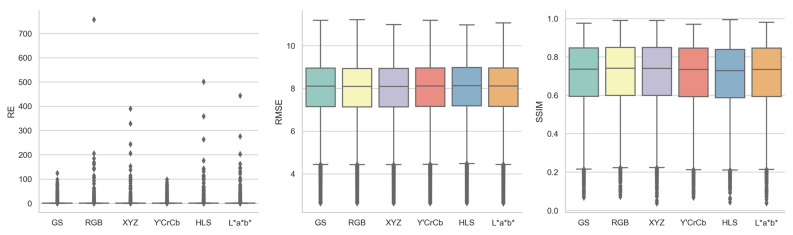
Boxplots of reprojection error (RE), root mean square error (RMSE), and structural similarity index measure (SSIM) achieved by each color space for all image registrations.

Figure 5, supplemented by Table A2, shows the boxplots of the three registration quality metrics achieved by each three-channel color space for all the image registrations, in comparison to one-channel grayscale (referred to as GS in the following figures). Overall, no color spaces differentiated themselves from others in a substantial way, regardless of the evaluation metrics, indicating that the utilization of image features from all three color channels did not bring obvious registration quality benefits. Based on the median values of the distributions, grayscale had a lower RE of 1.0005 than any other color spaces, which was likely due to its lower number of matched features coming from only one channel instead of all three channels, an RMSE of 8.1125, and an SSIM of 0.7363. For both RMSE and SSIM, RGB and XYZ consistently outperformed grayscale marginally, with RMSEs of 8.0981 and 8.0936 and SSIMs of 0.7410 and 0.7409, while Y′CrCb, HLS, and L*a*b* consistently underperformed grayscale marginally, with RMSEs of 8.1190, 8.1360, and 8.1159 and SSIMs of 0.7349, 0.7287, and 0.7354. Among the five color spaces, HLS seemed to be the least ideal one, with the largest RE and RMSE and the smallest SSIM.

**Figure 6:**
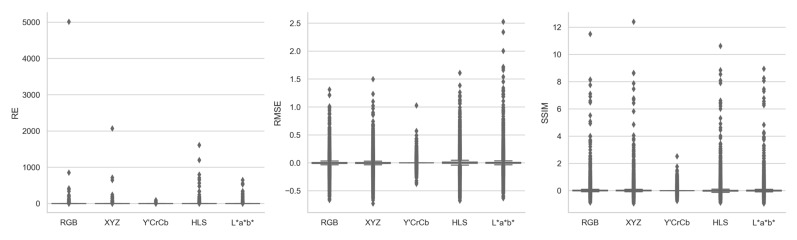
Boxplots of RE, RMSE, and SSIM relative change achieved by each color space over grayscale for all image registrations.

Figure 6, supplemented by Table A3, shows the boxplots of the registration quality relative changes achieved by each color space over grayscale, when their raw image pairs, feature detectors, and feature descriptors were identical. Generally, based on the median values of the distributions, no color space was able to improve RE over grayscale, likely for the reason mentioned above. However, again, RGB and XYZ both were able to improve RMSE and SSIM over grayscale marginally by 0.05% and 0.1%, indicating an expected image registration quality benefit when switching from grayscale to RGB or XYZ as input image channels. Overall Y′CrCb, as well as L*a*b* to a lesser degree, achieved an almost identical performance to grayscale with 0 or near 0 RE, RMSE, and SSIM relative changes. HLS again showed a consistently lower performance than grayscale, with median 10.57% RE increase, 0.09% RMSE increase, and 0.25% SSIM decrease.

Focusing on the outliers of the distributions in Figure 6, with the right raw image pair, feature detector, and feature descriptor combinations, all color spaces were able to either reduce image registration quality of grayscale by up to 9343% to 501,135% for RE, 103% to 253% for RMSE, and 76% to 95% for SSIM, or improve image registration quality of grayscale by up to 100% for RE, 37% to 73% for RMSE, and 252% to 1240% for SSIM. In that sense, no color space, including grayscale, is superior to others at all times, depending on the input image characteristics. Such large relative change ranges indicated the necessity of large benchmarking datasets for comparative image registration studies, as investigations based on only a few pair of outlier images could very likely result in misleading observations and conclusions.

#### 3.1.2. Color Channel

**Figure 7:**
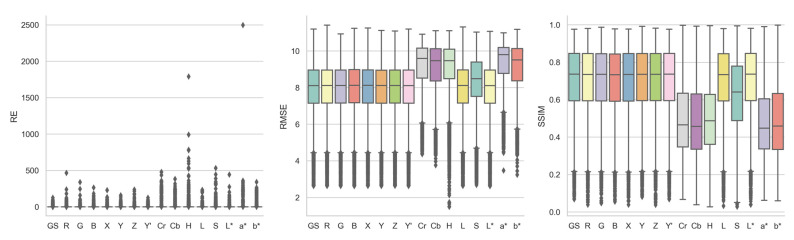
Boxplots of RE, RMSE, and SSIM achieved by each color channel for all image registrations.

Figure 7, supplemented by Table A4, shows the boxplots of the three registration quality metrics achieved by each individual color channel for all the image registrations, in comparison to grayscale. Overall, no color channels differentiated themselves from others in a meaningful positive way, although apparent inferior performances were observed for certain color channels. Based on RMSE and SSIM, Cr, Cb, H, S, a*, and b* were noticeably less accurate than the remaining color channels, all of which had similar performances to each other. In terms of RE median values, Cr, Cb, a*, and b* are much lower than the other color channels. Their 0 or near 0 first quartile RE values also indicated that they were not able to provide rich image features. Y′ and L* are the only two channels that outperformed grayscale based on RMSE and SSIM; however, their median values are almost identical to grayscales, with 8.1120 and 8.1122 versus 8.1125 for RMSE and 0.7364 and 0.7364 versus 0.7363 for SSIM. Note the calculation of Y′ channel should be the same as grayscale in theory, yet the function implementations in OpenCV occasionally resulted in minor image pixel value differences due to internal code base issues, leading to the trivial registration quality metric distribution differences between them.

**Figure 8:**
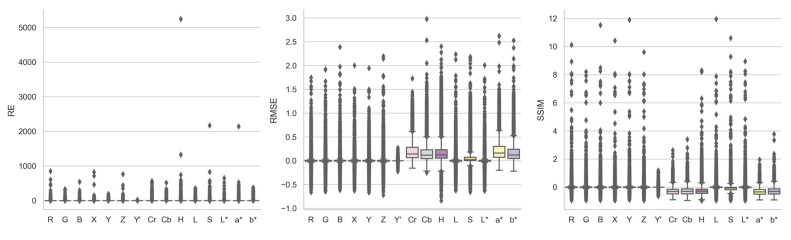
Boxplots of RE, RMSE, and SSIM relative change achieved by each color channel over grayscale for all image registrations.

Figure 8, supplemented by Table A5, shows the boxplots of the registration quality relative changes achieved by each color channel over grayscale, when their raw image pairs, feature detectors, and feature descriptors were identical. Based on the median values of the distributions, X, Cr, Cb, a*, and b* were able to achieve lower REs than grayscale, with a 0.43% to 54.24% reduction. L* was the only color channel that attained superior RMSE and SSIM to grayscale, with a marginal 0.01% improvement for both metrics. Cr, Cb, H, S, a*, and b* again showed substantially inferior performance to grayscale according to RMSE and SSIM, with an increase of 2.33% to 16.11% for RMSE and a decrease of 6.77% to 33.28% for SSIM.

Focusing on the outliers of the distributions in Figure 8, with the right raw image pair, feature detector, and feature descriptor combinations, similar to the color space observations, all color channels were able to either reduce image registration quality of grayscale by up to 2609% to 524,210% for RE, 27% to 297% for RMSE, and 64% to 96% for SSIM, or improve image registration quality of grayscale by up to 89 to 100% for RE, 16% to 72% for RMSE, and 119% to 1196% for SSIM. Again, no color channel, including grayscale, is superior to others at all times, depending on the input image characteristics. Relatively speaking, three-channel color spaces seemed to provide slight advantages over single-channel color channels in terms of improving the quality of the outlier grayscale registrations.

#### 3.1.3. Feature Detector

**Figure 9:**
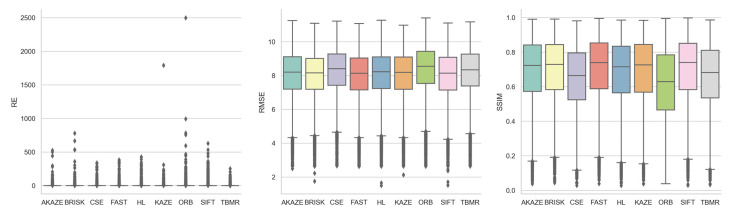
Boxplots of RE, RMSE, and SSIM achieved by each feature detector for all image registrations.

Figure 9, supplemented by Table A6, shows the boxplots of the three registration quality metrics achieved by each feature detector for all the image registrations. Based on the median RE values, from the best to the worst, the detectors ranked as AKAZE, SIFT, CSE, KAZE, HL, ORB, BRISK, FAST, and TBMR, with REs from 0.88 to 1.12. Based on the median RMSE and SSIM values, however, which mostly agreed with each other, from the best to the worst, the detectors ranked as FAST/SIFT, SIFT/FAST, BRISK, KAZE, AKAZE, HL, TBMR, CSE, and ORB, with RMSEs from 8.14 to 8.55 and SSIMs from 0.63 to 0.74. SIFT stood out as the most consistent-performing detector across all three metrics, securing second place in RE and RMSE and first place in SSIM.

#### 3.1.4. Feature Descriptor

**Figure 10:**
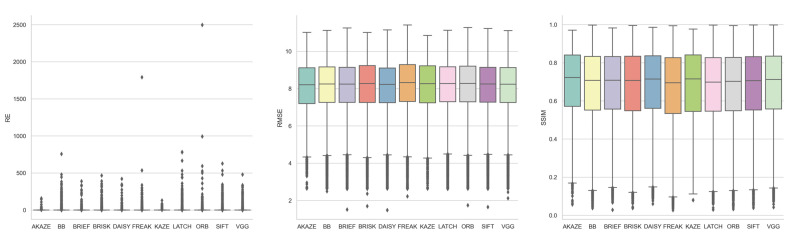
Boxplots of RE, RMSE, and SSIM achieved by each feature descriptor for all image registrations.

Figure 10, supplemented by Table A7, shows the boxplots of the three registration quality metrics achieved by each feature descriptor for all the image registrations. The descriptors did not differentiate themselves from each other as much as the detectors. Based on the median RE values, from the best to the worst, the descriptors ranked as AKAZE, KAZE, FREAK, BRISK, ORB, DAISY, BB, BRIEF, VGG, SIFT, and LATCH, with REs from 0.88 to 1.08. Based on the median RMSE values, from the best to the worst, the descriptors ranked as AKAZE, DAISY, VGG, BRIEF, SIFT, BB, KAZE, BRISK, LATCH, ORB, and FREAK, with RMSEs from 8.21 to 8.33. Based on the median SSIM values, from the best to the worst, the descriptors ranked as AKAZE, KAZE, DAISY, VGG, BRIEF, BB, BRISK, SIFT, ORB, LATCH, and FREAK, with SSIMs from 0.70 to 0.72. AKAZE consistently stood out as the best-performing descriptor across all three metrics. However, as shown in the upcoming section, AKAZE was one of the two descriptors with poor detector compatibility and hence high registration failure rate. The observed superior performance of AKAZE could be due to the lower influence from fewer low-performing detectors.

### 3.2. Registration Quality Metric Agreement

**Figure 11:**
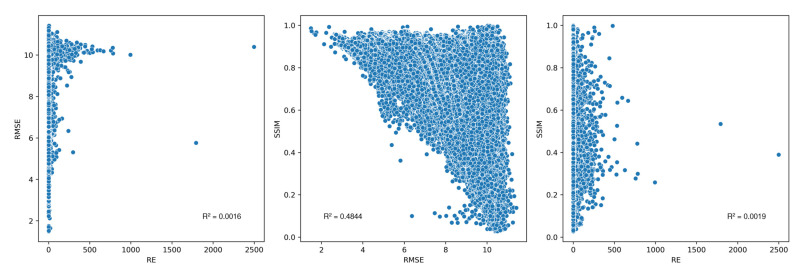
Scatter plots between RE, RMSE, and SSIM of all image registrations.

Figure 11 shows the scatter plots between the three registration quality metrics of all the image registrations. RMSE and SSIM were poorly correlated with RE, with coefficients of determination (R^2^s) of merely 0.0016 and 0.0019, respectively. This once again suggested the downside of RE as an image registration quality metric, potentially being extremely large for inaccurate registrations, unlike RMSE and SSIM, whose values fluctuated with narrow ranges. Additionally, as mentioned before, low REs could be simply the result of low matched feature numbers and did not guarantee accurate image registrations. RMSE and SSIM were better correlated, demonstrating a general negative correlation with a 0.4844 R^2^. Under the context of the study, which employed the unimodal dataset UDIS-D, RMSE seemed to be a superior and more reliable metric than SSIM. When RMSEs were low, such as being near 2, the corresponding SSIMs were also high, such as being near 1. However, when SSIMs were high, such as being near 1, the corresponding RMSEs distributed over the entire data range, such as being anywhere in between 2 and 11. In that regard, high-quality registrations identified through their RMSEs would likely also have high SSIMs, while high-quality registrations identified through their SSIMs would not necessarily have low RMSEs. Nevertheless, in terms of multimodal image registrations where image pixel values differ significantly, such as registrations between magnetic resonance imaging (MRI), computed tomography (CT), single-photon emission computed tomography (SPECT), positron emission tomography (PET), and ultrasound (US) images [72,73,74], or between optical, infrared, SAR, depth, map, day, and night images [75,76,77], SSIM might provide an advantage over RMSE to better quantify the similarity between registered target and source images.

### 3.3. Registration Failure Rate

**Figure 12:**
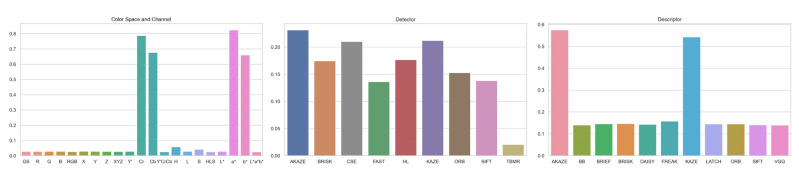
Bar charts for registration failure rates of the investigated color spaces and channels, feature detectors, and feature descriptors.

Figure 12 shows the registration failure rates of the investigated color channels and spaces, feature detectors, and feature descriptors respectively for all the image registrations. Cr, Cb, a*, and b* were the four color channels with very high failure rates, ranging from 66% to 82%. Figure 2 demonstrates a clear example showing their lack of image contrast relative to the other color channels and spaces, which could cause low numbers of detectable features. H and S also had noticeably high failure rates of 6% and 4%. From the best to the worst, the rest color channels and spaces ranked as L*a*b*, Y′CrCb, HLS, RGB, XYZ, grayscale, R, Z, L*, G, L, Y′, Y, X, and B, with failure rates varying from 2% to 3%. Interestingly, even though by a marginal difference, the five color spaces were more robust than any single image channels, including grayscale. From the best to the worst, the feature detectors ranked as TBMR, FAST, SIFT, ORB, BRISK, HL, CSE, KAZE, and AKAZE. Aside from TBMR, which had an unusually low failure rate of 2%, the failure rates of the rest detectors ranged from 14% to 23%. In terms of feature descriptor, AKAZR and KAZE were the two with abnormally high failure rates of 57% and 54%, mostly due to their frequent incompatibility with most feature detectors. From the best to the worst, the rest descriptors ranked as VGG, BB, SIFT, DAISY, LATCH, ORB, BRIEF, BRISK, and FREAK, with failure rates ranging from 14% to 16%.

### 3.4. Feature Number

#### 3.4.1. Color Channel

**Figure 13:**
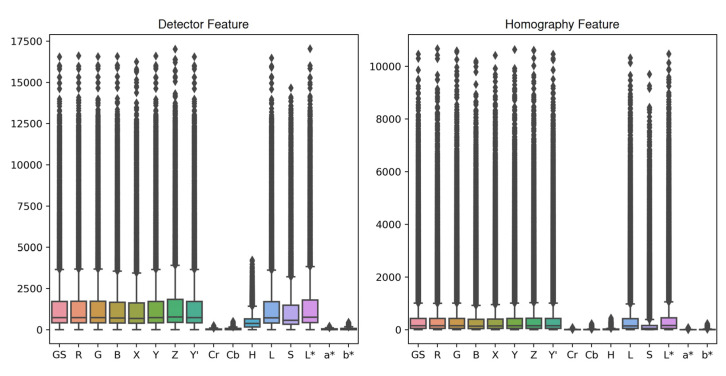
Boxplots of initial detector feature numbers and final homography feature numbers achieved by each color channel for all image registrations.

Figure 13, supplemented by Table A8, shows the distributions of the initial feature numbers in the target and source images detected by the feature detectors, as well as the inlier matched feature numbers in the target or source images after RANSAC used for homography estimation, achieved by each color channel for all the image registrations. Based on the distribution median values, as expected, Cr, Cb, a*, and b* had very low numbers of initial detectable features and final inlier features, with 24 to 31 detector features and 6 to 7 homography features. H and S also had considerably lower features than the most color channels, with 390 and 575 detector features and 10 and 53 homography features. From the most to the least, the rest of the color channels ranked as Z, L*, R, G, Y, Y′, grayscale, L, B, and X, with 399 to 449 detector features, and L*, G/Y/Y′/grayscale, Z/R, L, X, and B, with 144 to 162 homography features. As the best performing color channel in terms of registration quality, L* also ranked at the top in terms of feature numbers with the second-most initial detectable features and the most final inlier features. On the other hand, Cr, Cb, H, S, a*, and b* not only attained the lowest registration quality, but also had the lowest detector and homography features. This observation indicated the potential positive association between image feature number and image registration quality. In that sense, artificial intelligence-based image contrast enhancement and resolution upscaling might be a future research direction for improving image registration accuracy.

#### 3.4.2. Feature Detector

**Figure 14:**
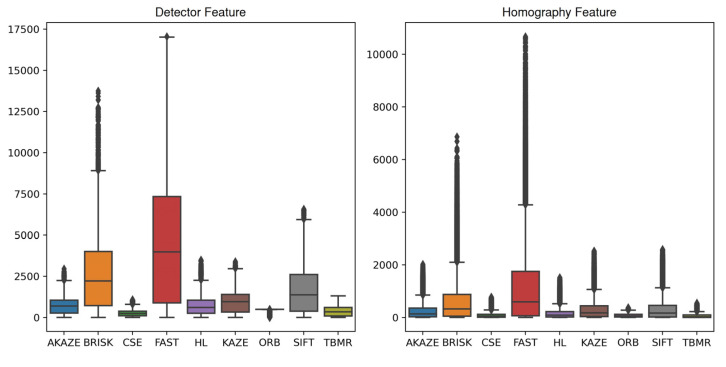
Boxplots of initial detector feature numbers and final homography feature numbers achieved by each feature detector for all image registrations.

Figure 14, supplemented by Table A9, shows the distributions of the initial feature numbers in the target and source images detected by the feature detectors, as well as the inlier matched feature numbers in the target or source images after RANSAC used for homography estimation, achieved by each feature detector for all the image registrations. Substantial feature number differences were observed for the detectors. Based on the distribution median values, the detector rankings for the initial and final feature numbers mostly agreed with each other, being FAST, BRISK, SIFT/KAZE, KAZE/SIFT, AKAZE, HL, ORB, TBMR/CSE, and CSE/TBMR, from the most to the least. The initial detector feature numbers ranged from 3988 to 244, while the final homography feature numbers ranged from 600 to 33. FAST, as the one of the two best-performing detectors based on RMSE and SSIM, provided significantly more features than the remaining detectors, surpassing the second-place detector, BRISK, by 80% and 83% in terms of initial detector and final homography features. Again, the potential association between image feature number and image registration quality was observed. Aside from image registration, image features also have applications in object recognition [78], object detection [79], image retrieval [80], 3D reconstruction [81], etc., which all might benefit from the large image feature numbers identified by detectors such as FAST, allowing for richer representations of objects and more potential feature correspondences.

#### 3.4.3. Feature Descriptor

**Figure 15:**
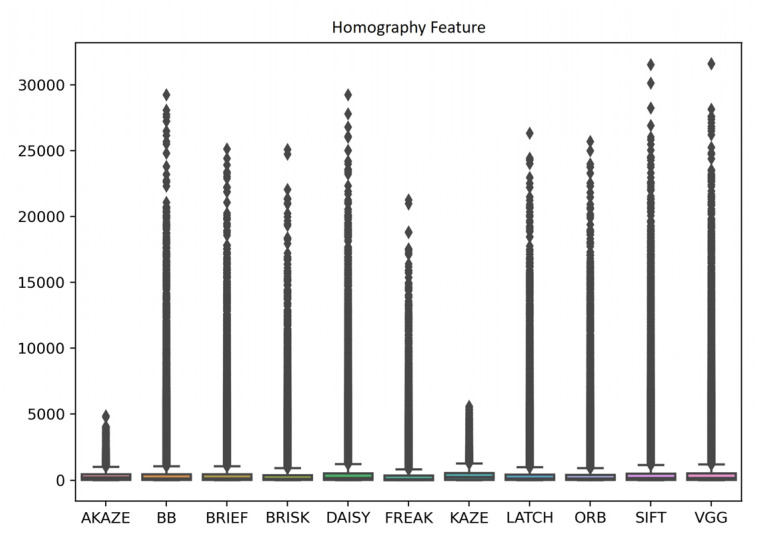
Boxplots of initial detector feature numbers and final homography feature numbers achieved by each feature descriptor for all image registrations.

Figure 15, supplemented by Table A10, shows the distributions of the inlier matched feature numbers in the target or source images after RANSAC used for homography estimation, achieved by each feature descriptor for all the image registrations. No significant feature number differences between the descriptors were observed unlike the detectors, indicating feature detector was potentially a bigger factor than feature descriptor in regard to influencing the numbers of final inlier homography features. Based on the distribution median values, from the most to the least, the descriptors ranked as KAZE, AKAZE, DAISY, VGG, SIFT, BRIEF, LATCH, BB, ORB, BRISK, and FREAK, with homography feature numbers ranging from 87 to 215.

### 3.5. Best Color Space or Channel, Detector, and Descriptor Combination

**Figure 16:**
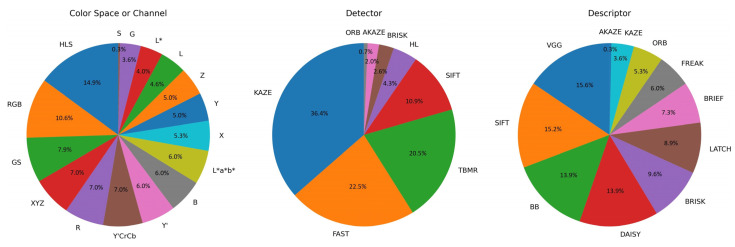
Composition pie charts of the 302 color space or channel, feature detector, and feature descriptor combinations that registered the entire dataset without failure.

Due to the large number of combinations, the selection of the best color space or color channel, feature detector, and feature descriptor combinations was based on one prerequisite: the selected combinations shall never fail for any image registrations. Out of the 21 color spaces or channels × 84 detector and descriptor combinations = 1764 total combinations, each of which performed 1106 registrations, 302 or 17% of them successfully registered all the images in the dataset without failure. Figure 16 shows the composition pie charts of the 302 unfailing combinations in terms of color space or channel, feature detector, and feature descriptor. Among the 21 investigated color spaces or channels, Cr, Cb, H, a* and b* always failed at least once when registering the entire dataset, regardless of their paired detectors and descriptors. Interestingly, HLS had the highest proportion of unfailing combinations than any other color spaces or channels. Among the nine investigated feature detectors, only CSE was not able to register all the dataset images without failure. On the other hand, with the right color spaces or channels and detectors, all 11 investigated feature descriptors were able to achieve successful registrations for all the dataset images.

In terms of average values over the entire dataset, for the 302 unfailing combinations, their REs ranged from 0.86 to 1.60, their RMSEs ranged from 7.80 to 8.30, and their SSIMs ranged from 0.63 to 0.75. The top 10 combinations for each metric below can be found in Table A11. As the combinations with consecutive placing according to any of the three registration quality metrics often had very small differences, the following color space or channel, detector, and descriptor combination recommendations were strictly based on and confined by the UDIS-D dataset:

- Lowest RE combinations
  For color space, XYZ+KAZE+BRISK ranked at 2nd place, with an RE of 0.86, an RMSE of 7.85 at 102nd place, and an SSIM of 0.73 at 102nd place. For color channel, L+KAZE+BRISK ranked at 1st place, with an RE of 0.86, an RMSE of 7.88 at 166th place, and an SSIM of 0.73 at 153rd place.
- Lowest RMSE combinations
  For color space, RGB+SIFT+VGG ranked at 1st place, with an RMSE of 7.80, an RE of 0.90 at 21st place, and an SSIM of 0.74 at 4th place. For color channel, Y′+FAST+VGG, which should be equivalent to grayscale+FAST+VGG, ranked at 7th place, with an RMSE of 7.81, an RE of 1.15 at 181st place, and an SSIM of 0.74 at 6th place.
- Highest SSIM combinations
  For color space, XYZ+SIFT+SIFT ranked at 1st place, with an SSIM of 0.75, an RE of 0.90 at 18th place, and an RMSE of 7.80 at 2nd place. For color channel, G+FAST+VGG ranked at 5th place, with an SSIM of 0.74, an RE of 1.15 at 184th place, and an RMSE of 7.81 at 12th place.
- Most detector feature combinations
  For color channel, Z+FAST+VGG ranked at 39th place, with a detector feature number of 11,642, and a homography feature number of 1960 at 21st place.
- Most homography feature combinations
  For color channel, Z+FAST+VGG ranked at 21st place, with a homography feature number of 1960, and a detector feature number of 11,642 at 39th place, as mentioned above.

## 4. Conclusions

The following conclusions were made strictly based on the UDIS-D dataset and only applicable to the investigated color spaces and channels, feature detectors, and feature descriptors, without considering the incompatible detector and descriptor combinations.

From an atomistic point of view, two color spaces, XYZ and RGB, as well as one color channel, L*, provided very minor image registration quality improvement over grayscale. SIFT, and potentially FAST, were the best-performing detectors. AKAZE was the best-performing descriptor. L*a*b* was the most robust color space, and grayscale was the most robust color channel. TBMR was the most robust detector. VGG was the most robust descriptor. Z channel allowed the most initial detector features, while L* channel allowed the most final homography features. FAST detector provided the most detector and homography features, while KAZE descriptor provided the most homography features.

From a holistic point of view, color space XYZ and RGB, detector SIFT and FAST, and descriptor VGG and SIFT seemed to optimize RMSE and SSIM the most. The KAZE detector and BRISK descriptor combination seemed to provide special benefits for optimizing RE. The Z channel, FAST detector, and VGG descriptor combination allowed for the detection of the most initial detector features as well as the preservation of the most final homography features.

## 5. Feature Acronym

The extended forms of the image feature detectors and descriptors mentioned in this article include:

- AGAST: adaptive and generic accelerated segment test
- AKAZE: accelerated-KAZE
- ASIFT: affine-SIFT
- BB: BinBoost
- BEBLID: boosted efficient binary local image descriptor
- BRIEF: binary robust independent elementary features
- BRISK: binary robust invariant scalable keypoints
- CSE: center surround extremas
- CSIFT: colored SIFT
- CURVE: local feature of retinal vessels
- FAST: features from accelerated segment test
- FREAK: fast retina keypoint
- GFTT: good features to track
- GOFRO: Gabor odd filter ratio-based operator
- GSIFT: global context SIFT
- HC: Harris corner
- HL: Harris–Laplace
- HOG: histograms of oriented gradient
- LATCH: learned arrangements of three patch codes
- LUCID: locally uniform comparison image descriptor
- MSD: maximal self-dissimilarities
- MSER: maximally stable extremal regions
- ORB: oriented FAST and rotated BRIEF
- PCA-SIFT: principal components analysis-SIFT
- PCT: position–color–texture
- SIFT: scale invariant feature transform
- SQFD: signature quadratic form distance
- SURF: speeded up robust features
- TBMR: tree-based Morse regions
- TEBLID: triplet-based efficient binary local image descriptor
- VGG: Visual Geometry Group

## Figures and Tables

**Table 1:** Selected image feature detectors for the study.

Feature Detector	Reference	OpenCV Initialization Function
AKAZE	[25]	cv2.AKAZE_create()
BRISK	[26]	cv2.BRISK_create()
CSE	[27]	cv2.xfeatures2d.StarDetector_create()
FAST	[28]	cv2.FastFeatureDetector_create()
HL	[29]	cv2.xfeatures2d.HarrisLaplaceFeatureDetector_create()
KAZE	[30]	cv2.KAZE_create()
ORB	[32]	cv2.ORB_create()
SIFT	[33]	cv2.SIFT_create()
TBMR	[35]	cv2.xfeatures2d.TBMR_create()

**Table 2:** Selected image feature descriptors for the study.

Feature Descriptor	Reference	OpenCV Initialization Function
AKAZE	[25]	cv2.AKAZE_create()
BB	[36]	cv2.xfeatures2d.BoostDesc_create()
BRIEF	[38]	cv2.xfeatures2d.BriefDescriptorExtractor_create()
BRISK	[26]	cv2.BRISK_create()
DAISY	[39]	cv2.xfeatures2d.DAISY_create()
FREAK	[36]	cv2.xfeatures2d.FREAK_create()
KAZE	[30]	cv2.KAZE_create()
LATCH	[41]	cv2.xfeatures2d.LATCH_create()
ORB	[32]	cv2.ORB_create()
SIFT	[33]	cv2.SIFT_create()
VGG	[46]	cv2.xfeatures2d.VGG_create()

## Data Availability

The dataset UDIS-D is publicly accessible through the link provided by the dataset authors.

## Appendix A

This section provides detailed information not shown in the main article.

**Table A1:** Incompatible OpenCV image feature detectors and descriptors.

Feature Detector	Feature Descriptor
BRISK	AKAZE
BRISK	KAZE
CSE	AKAZE
CSE	KAZE
FAST	AKAZE
FAST	KAZE
HL	AKAZE
HL	KAZE
ORB	AKAZE
ORB	KAZE
SIFT	AKAZE
SIFT	KAZE
SIFT	ORB
TBMR	AKAZE
TBMR	KAZE

**Table A2:** Image registration quality metric distributions of the investigated color spaces.

Color Space	RE					RMSE					SSIM				
	Min	Q1	Median	Q3	Max	Min	Q1	Median	Q3	Max	Min	Q1	Median	Q3	Max
GS	0	0.8024	1.0005	1.2121	124.2114	2.6329	7.1536	8.1125	8.9581	11.2000	0.0679	0.5946	0.7363	0.8472	0.9767
RGB	5.80 × 10^−14^	0.8328	1.0282	1.2357	757.4403	2.6369	7.1372	8.0981	8.9335	11.2248	0.0721	0.5993	0.7410	0.8500	0.9905
XYZ	2.99 × 10^−14^	0.8229	1.0196	1.2288	389.9551	2.6332	7.1390	8.0936	8.9411	10.9971	0.0374	0.5994	0.7409	0.8500	0.9910
Y′CrCb	2.94 × 10^−14^	0.8055	1.0081	1.2200	97.7295	2.6329	7.1598	8.1190	8.9635	11.2000	0.0679	0.5932	0.7349	0.8466	0.9707
HLS	3.08 × 10^−14^	0.9268	1.1179	1.3088	500.9235	2.6434	7.1856	8.1360	8.9832	10.9812	0.0428	0.5878	0.7287	0.8392	0.9948
L*a*b*	2.89 × 10^−14^	0.8104	1.0132	1.2254	443.2364	2.6271	7.1579	8.1195	8.9659	11.0763	0.0387	0.5935	0.7354	0.8465	0.9814

**Table A3:** Image registration quality relative change distribution of the investigated color spaces over grayscale.

Color Space	RE					RMSE					SSIM				
	Min	Q1	Median	Q3	Max	Min	Q1	Median	Q3	Max	Min	Q1	Median	Q3	Max
RGB	−1	−0.0312	0.0197	0.0822	5011.3504	−0.6645	−0.0098	−0.0005	0.0071	1.3114	−0.8751	−0.0189	0.0010	0.0268	11.5030
XYZ	−1	−0.0360	0.0135	0.0714	2071.0455	−0.7286	−0.0096	−0.0005	0.0069	1.4980	−0.9434	−0.0181	0.0010	0.0262	12.3977
Y′CrCb	−1	−7.25 × 10^−7^	7.01 × 10^−8^	2.84 × 10^−6^	93.4272	−0.3746	−5.14 × 10^−11^	0	5.28 × 10^−11^	1.0278	−0.7606	−5.84 × 10^−10^	0	5.64 × 10^−10^	2.5220
HLS	−1	0.0209	0.1057	0.2198	1610.0101	−0.6761	−0.0083	0.0009	0.0134	1.6110	−0.8839	−0.0359	−0.0025	0.0218	10.6199
L*a*b*	−1	−0.0451	0.0106	0.0717	648.5143	−0.6329	−0.0085	1.14 × 10^−5^	0.0087	2.5251	−0.9523	−0.0235	−2.36 × 10^−5^	0.0234	8.9460

**Table A4:** Image registration quality metric distributions of the investigated color channels.

Color Channel	RE					RMSE					SSIM				
	Min	Q1	Median	Q3	Max	Min	Q1	Median	Q3	Max	Min	Q1	Median	Q3	Max
GS	0	0.8024	1.0005	1.2121	124.2114	2.6329	7.1536	8.1125	8.9581	11.2000	0.0679	0.5946	0.7363	0.8472	0.9767
R	0	0.8142	1.0112	1.2181	466.5051	2.6300	7.1577	8.1187	8.9625	11.4128	0.0391	0.5939	0.7350	0.8467	0.9808
G	0	0.8059	1.0024	1.2144	339.3203	2.6346	7.1558	8.1172	8.9629	10.9353	0.0473	0.5935	0.7355	0.8461	0.9873
B	0	0.8184	1.0176	1.2218	264.9574	2.6259	7.1747	8.1271	8.9807	11.2438	0.0549	0.5916	0.7330	0.8439	0.9781
X	0	0.7970	0.9966	1.2076	230.0495	2.6380	7.1618	8.1233	8.9696	11.2540	0.0392	0.5930	0.7348	0.8461	0.9771
Y	0	0.8024	1.0022	1.2126	158.1726	2.6340	7.1537	8.1154	8.9556	11.1215	0.0810	0.5948	0.7357	0.8469	0.9922
Z	0	0.8229	1.0217	1.2262	238.1934	2.6443	7.1611	8.1156	8.9599	11.0945	0.0377	0.5948	0.7354	0.8462	0.9824
Y′	0	0.8024	1.0009	1.2122	124.2114	2.6329	7.1533	8.1120	8.9572	11.2000	0.0679	0.5948	0.7364	0.8473	0.9767
Cr	0	0	0.5328	0.9075	479.0374	4.3705	8.5232	9.5965	10.1616	10.9218	0.0668	0.3479	0.4656	0.6348	0.9979
Cb	0	0.0301	0.6558	0.9893	383.1054	3.7606	8.3612	9.4740	10.1213	11.1037	0.0386	0.3359	0.4576	0.6306	0.9931
H	0	0.7259	1.1107	1.4043	1791.3949	1.4912	8.4892	9.4695	10.0998	11.1104	0.0274	0.3612	0.4878	0.6279	0.9954
L	0	0.8073	1.0051	1.2138	232.3559	2.6425	7.1604	8.1183	8.9682	11.3127	0.0325	0.5932	0.7343	0.8460	0.9796
S	0	0.9138	1.1271	1.3139	532.2115	2.6888	7.5192	8.4918	9.3998	11.0361	0.0282	0.4880	0.6414	0.7797	0.9942
L*	0	0.8072	1.0061	1.2169	443.2365	2.6271	7.1543	8.1122	8.9584	11.0763	0.0387	0.5954	0.7364	0.8471	0.9814
a*	0	0	0.4561	0.8876	2497.2551	3.4712	8.7800	9.8045	10.1946	10.9912	0.0619	0.3376	0.4480	0.6051	0.9903
b*	0	0	0.6189	0.9804	341.4606	3.2459	8.3756	9.5108	10.1313	11.1813	0.0601	0.3340	0.4592	0.6334	0.9984

**Table A5:** Image registration quality relative change distribution of the investigated color channels over grayscale.

Color Channel	RE					RMSE					SSIM				
	Min	Q1	Median	Q3	Max	Min	Q1	Median	Q3	Max	Min	Q1	Median	Q3	Max
R	−1	−0.0477	0.0111	0.0741	852.7151	−0.6608	−0.0085	0.0001	0.0092	1.7467	−0.9391	−0.0249	−0.0002	0.0232	10.1168
G	−1	−0.0511	0.0044	0.0615	332.1132	−0.6492	−0.0082	0.0001	0.0088	1.9223	−0.9366	−0.0235	−0.0002	0.0225	8.2042
B	−1	−0.0481	0.0172	0.0902	542.3107	−0.6152	−0.0080	0.0006	0.0110	2.3887	−0.9221	−0.0293	−0.0012	0.0219	11.5289
X	−1	−0.0579	−0.0043	0.0518	824.0254	−0.6296	−0.0078	0.0002	0.0088	1.9988	−0.9427	−0.0240	−0.0003	0.0214	10.4170
Y	−1	−0.0513	0.0009	0.0550	195.2386	−0.6662	−0.0081	1.82 × 10^−5^	0.0082	1.9427	−0.9080	−0.0220	−2.12 × 10^−5^	0.0219	11.9081
Z	−1	−0.0427	0.0213	0.0909	762.2179	−0.7218	−0.0087	0.0002	0.0097	2.1930	−0.9498	−0.0257	−0.0003	0.0242	9.6052
Y′	−0.8925	0	0	0	26.0947	−0.2221	−2.21 × 10^−11^	0	2.15 × 10^−11^	0.2683	−0.6383	−2.34 × 10^−10^	0	2.43 × 10^−10^	1.1874
Cr	−1	−1	−0.4585	−0.1018	561.2052	−0.1556	0.0625	0.1431	0.2829	1.7261	−0.8972	−0.4754	−0.3029	−0.1478	2.6270
Cb	−1	−0.9578	−0.3282	0.0032	516.4805	−0.2510	0.0458	0.1165	0.2286	2.9749	−0.9438	−0.4785	−0.2917	−0.1189	3.3976
H	−1	−0.2999	0.0795	0.5094	5242.1047	−0.8390	0.0531	0.1252	0.2327	2.4010	−0.9596	−0.4415	−0.2810	−0.1412	8.3001
L	−1	−0.0514	0.0060	0.0661	355.8259	−0.6256	−0.0082	0.0002	0.0094	2.2343	−0.9485	−0.0249	−0.0004	0.0221	11.9563
S	−1	−0.0491	0.1300	0.3220	2169.3907	−0.6589	0.0016	0.0233	0.0741	2.1806	−0.9463	−0.1875	−0.0677	−0.0081	10.6029
L*	−1	−0.0505	0.0054	0.0632	648.5143	−0.6329	−0.0086	−5.09 × 10^−5^	0.0084	2.0023	−0.9523	−0.0227	6.33 × 10^−5^	0.0236	8.9460
a*	−1	−1	−0.5424	−0.1581	2143.7176	−0.2017	0.0740	0.1611	0.3009	2.6181	−0.9073	−0.4924	−0.3328	−0.1660	1.9590
b*	−1	−1	−0.3608	−0.0089	381.0833	−0.2206	0.0491	0.1218	0.2401	2.5251	−0.9027	−0.4818	−0.2973	−0.1231	3.7708

**Table A6:** Image registration quality metric distributions of the investigated feature detectors.

Feature Detector	RE					RMSE					SSIM				
	Min	Q1	Median	Q3	Max	Min	Q1	Median	Q3	Max	Min	Q1	Median	Q3	Max
AKAZE	0	0.6568	0.8834	1.1340	524.5237	2.4982	7.1991	8.2058	9.1134	11.2540	0.0377	0.5728	0.7238	0.8419	0.9910
BRISK	0	0.8745	1.0778	1.3006	778.7086	1.7508	7.1897	8.1602	9.0154	11.0945	0.0436	0.5830	0.7298	0.8442	0.9922
CSE	0	0.7422	0.9207	1.1453	339.3203	2.6642	7.4287	8.4110	9.2794	11.2248	0.0282	0.5245	0.6651	0.7958	0.9814
FAST	0	0.9257	1.1112	1.3154	383.1054	2.6259	7.1557	8.1359	9.0392	11.0833	0.0389	0.5887	0.7400	0.8538	0.9954
HL	0	0.8584	1.0380	1.2472	428.2064	1.4912	7.2412	8.2302	9.1089	11.2796	0.0274	0.5648	0.7156	0.8343	0.9863
KAZE	0	0.6833	0.9225	1.1704	1791.3949	2.1254	7.1924	8.1953	9.1002	10.9844	0.0374	0.5686	0.7266	0.8451	0.9846
ORB	0	0.9023	1.0559	1.2350	2497.2551	2.6346	7.5405	8.5499	9.4351	11.4128	0.0390	0.4660	0.6298	0.7846	0.9948
SIFT	0	0.6747	0.8905	1.1284	627.4206	1.5188	7.1476	8.1495	9.0884	11.1104	0.0293	0.5832	0.7409	0.8518	0.9984
TBMR	0	0.8976	1.1194	1.3020	251.3074	2.6418	7.3862	8.3409	9.2696	11.1813	0.0325	0.5351	0.6823	0.8109	0.9866

**Table A7:** Image registration quality metric distributions of the investigated feature descriptors.

Feature Descriptor	RE					RMSE					SSIM				
	Min	Q1	Median	Q3	Max	Min	Q1	Median	Q3	Max	Min	Q1	Median	Q3	Max
AKAZE	0	0.6475	0.8802	1.1424	157.9498	2.6432	7.2020	8.2082	9.1180	11.0190	0.0567	0.5720	0.7232	0.8409	0.9712
BB	0	0.8129	1.0296	1.2451	757.4403	2.4982	7.2662	8.2538	9.1667	11.1215	0.0377	0.5520	0.7080	0.8333	0.9979
BRIEF	0	0.8244	1.0338	1.2487	389.1299	1.5188	7.2679	8.2460	9.1433	11.2540	0.0282	0.5576	0.7087	0.8327	0.9826
BRISK	0	0.7288	0.9284	1.1320	466.5051	1.7044	7.2612	8.2753	9.2346	11.0102	0.0374	0.5487	0.7077	0.8344	0.9954
DAISY	0	0.8032	1.0292	1.2534	421.1894	1.4912	7.2452	8.2260	9.1080	11.1505	0.0601	0.5615	0.7151	0.8369	0.9863
FREAK	0	0.7080	0.9016	1.1169	1791.3949	2.2251	7.3115	8.3258	9.2896	11.4128	0.0274	0.5344	0.6951	0.8270	0.9945
KAZE	0	0.6428	0.8981	1.1690	130.8570	2.6395	7.2435	8.2682	9.2238	10.8529	0.0795	0.5449	0.7155	0.8415	0.9771
LATCH	0	0.8625	1.0818	1.2958	783.5445	2.6259	7.3006	8.2769	9.1702	11.1286	0.0293	0.5462	0.6985	0.8272	0.9979
ORB	0	0.8233	1.0219	1.2167	2497.2551	1.7508	7.2953	8.2827	9.2057	11.2796	0.0325	0.5489	0.7027	0.8283	0.9948
SIFT	0	0.8459	1.0662	1.2871	627.4206	1.6517	7.2750	8.2512	9.1394	11.2248	0.0390	0.5529	0.7065	0.8324	0.9984
VGG	0	0.8326	1.0447	1.2584	479.0374	2.1254	7.2579	8.2365	9.1280	11.1104	0.0421	0.5581	0.7125	0.8351	0.9984

**Table A8:** Distributions of initial detector feature numbers and final homography feature numbers of the investigated color channels.

Color Channel	Detector Feature Number					Homography Feature Number				
	Min	Q1	Median	Q3	Max	Min	Q1	Median	Q3	Max
GS	4	420	735	1715	16,556	4	54	157	436	10,460
R	5	424	746	1728.75	16,611	4	53	155	432	10,670
G	5	425	740	1727	16,565	4	54	157	436	10,586
B	4	413	721	1669	16,575	4	49	144	400	10,191
X	4	399	695.5	1619	16,254	4	52	151	412	10,417
Y	4	422	737.5	1717.75	16,597	4	54	157	437	10,638
Z	4	449	778	1833.75	17,013	4	53	155	442	10,610
Y′	5	420	735.5	1715	16,556	4	54	157	437	10,460
Cr	4	11	24	51	260	4	4	6	11	86
Cb	4	13	31	79	512	4	5	7	15	236
H	4	167	390	667	4224	4	6	10	27	447
L	4	415	724.5	1695	16,481	4	52.25	152	423	10,324
S	4	337	575	1487	14,673	4	15	53	167	9701
L*	5	441	768.5	1799	17,047	4	56	162	457	10,474
a*	4	11	25	58	224	4	4	6	9	84
b*	4	12	29	80	453	4	4	7	14	237

**Table A9:** Distributions of initial detector feature numbers and final homography feature numbers of the investigated feature detectors.

Feature Detector	Detector Feature Number					Homography Feature Number				
	Min	Q1	Median	Q3	Max	Min	Q1	Median	Q3	Max
AKAZE	4	269	707	1063	2947	4	31	145	360	2021
BRISK	4	733	2219	4006	13746	4	58	328	877	6866
CSE	4	107	244	386	1073	4	13	52	124	771
FAST	4	893	3987.5	7341	17,047	4	69	600	1756	10,670
HL	4	256	618	1059	3488	4	26	97	227	1514
KAZE	4	341	964	1395	3394	4	36	177	448	2529
ORB	4	496	500	500	501	4	21	62	125	376
SIFT	4	389	1375	2613	6576	4	25	171	467	2588
TBMR	4	98	351	621	1310	4	9	33	98	551

**Table A10:** Distributions of final homography feature numbers of the investigated feature descriptors.

Feature Descriptor	Homography Feature Number				
	Min	Q1	Median	Q3	Max
AKAZE	4	44	191	429	4860
BB	4	32	133	440	29,264
BRIEF	4	37	140	437	25,130
BRISK	4	25	111	373	25,094
DAISY	4	43	161	505	29,265
FREAK	4	20	87	338	21,257
KAZE	4	38	215	525	5596
LATCH	4	34	135	407	26,330
ORB	4	30	127	380	25,698
SIFT	4	37	145	481	31,523
VGG	4	40	155	499	31,604

**Table A11:** Top 10 color space or channel, feature detector, and feature descriptor combinations in terms of average RE, RMSE, SSIM, detector feature number, and homography feature number over the entire dataset.

Place	RE		RMSE		SSIM		Detector Feature Number		Homography Feature Number	
	Combination	Value	Combination	Value	Combination	Value	Combination	Value	Combination	Value
1st	L+KAZE+BRISK	0.8626	RGB+SIFT+VGG	7.8020	XYZ+SIFT+SIFT	0.7467	Z+FAST+VGG	11,641.89	Z+FAST+VGG	1960.12
2nd	XYZ+KAZE+BRISK	0.8631	XYZ+SIFT+SIFT	7.8038	XYZ+SIFT+VGG	0.7453	Z+FAST+BB	11,641.89	L*+FAST+VGG	1925.72
3rd	RGB+SIFT+BRISK	0.8635	XYZ+SIFT+VGG	7.8056	RGB+SIFT+SIFT	0.7446	Z+FAST+BRISK	11,641.89	G+FAST+VGG	1885.80
4th	X+KAZE+FREAK	0.8639	RGB+FAST+VGG	7.8059	RGB+SIFT+VGG	0.7440	L*+FAST+VGG	11,175.85	Y+FAST+VGG	1884.00
5th	Y′CrCb+KAZE+BRISK	0.8660	RGB+FAST+DAISY	7.8078	G+FAST+VGG	0.7439	L*+FAST+DAISY	11,175.85	R+FAST+VGG	1875.15
6th	Y′+KAZE+BRISK	0.8702	Y′CrCb+FAST+VGG	7.8092	Y′+FAST+VGG	0.7436	L*+FAST+BRISK	11,175.85	Y′+FAST+VGG	1868.76
7th	Y+KAZE+BRISK	0.8704	Y′+FAST+VGG	7.8106	B+SIFT+SIFT	0.7435	L*+FAST+SIFT	11,175.85	GS+FAST+VGG	1867.36
8th	GS+KAZE+BRISK	0.8713	GS+FAST+VGG	7.8109	GS+FAST+VGG	0.7435	B+FAST+VGG	11,018.69	L*+FAST+SIFT	1855.61
9th	R+SIFT+BRISK	0.8725	R+FAST+VGG	7.8126	RGB+FAST+VGG	0.7434	B+FAST+DAISY	11,018.69	B+FAST+VGG	1848.31
10th	RGB+SIFT+BB	0.8744	Z+FAST+VGG	7.8127	Y+FAST+VGG	0.7433	B+FAST+BRISK	11,018.69	X+FAST+VGG	1826.52
